# How happenings do (not) turn into events: A typology and an application to the case of 9/11 in the American and Dutch public spheres

**DOI:** 10.1111/1468-4446.12847

**Published:** 2021-05-06

**Authors:** Thijs van Dooremalen

**Affiliations:** ^1^ Weatherhead Center for International Affairs Harvard University Cambridge MA USA

**Keywords:** 9/11, cleavage structures, cultural repertoires, discursive opportunities, events

## Abstract

Why do some happenings become incentives for cultural or political transformation (that is: turn into events), whereas others remain ordinary occurrences? The theoretical perspectives of cultural repertoires, cleavage structures, and discursive opportunities are prominent and fruitful approaches for explaining cultural or political behavior and attitudes, yet they do not have a satisfactory answer to this question. To fill in this gap, I introduce a typology that indicates how certain happenings merely reproduce existing trends, whereas other ones turn into motives to change them. This can be either because they are “focus events,” which confirm dominant cultural or political patterns, or because they are “shock events,” which form a break from them. I illustrate this typology by investigating the distinct meanings that 9/11 were accorded in the American and Dutch public spheres. This analysis shows that this happening became a “shock event” on the issue of safety in the American case, as it broke with the cultural repertoire of viewing the United States as a safe, militarily impenetrable nation. In contrast, 9/11 turned into a “focus event” concerning the issue of Islam in the Dutch case because it confirmed the discursive opportunities to problematize Muslims, which public actors in the Netherlands had already developed in the years leading up to 2001.

## 1 INTRODUCTION

In recent decades, various perspectives have been developed within the fields of sociology and political science for making cross‐national comparisons of cultural or political behavior and attitudes. Among the most fruitful and prominent are “cultural repertoires” (e.g., Lamont, 1992, 2000, 2019; Lamont et al., 2016; Lamont & Thévenot, 2000), “cleavage structures” (e.g., Kriesi et al., 1995, 2008; Kriesi & Pappas, 2015), and “discursive opportunity structures” (e.g., Koopmans & Olzak, 2004; Koopmans & Statham, 2010; Koopmans et al., 2005; Ushiyama, 2019). Today, many sociologists and political scientists use one of these perspectives when engaging in country‐comparative research.

However, what is missing in each of these perspectives is a satisfactory answer to the question of how social change can come about, specifically in response to events. To what extent do events like 9/11, the Brexit referendum, or the Trump election offer the possibility to transform the given cultural repertoires, cleavage structures, and discursive opportunities? Events often remain a black box in each of the perspectives. Either they are presented as occasions that simply reproduce long‐term trends (e.g., Koopmans & Olzak, 2004; Lamont et al., 2016) or they are conceptualized as shocking happenings that spontaneously create a radical break with existing traditions (e.g., Kriesi & Pappas, 2015; Lamont, 1992). A serious theoretical reflection on the mechanisms of when events come about and how they can be transformative is usually absent.

The aim of this paper is to fill in this theoretical gap by relating the transformative potential of events to these three perspectives. I propose that this can be done through conceptualizing events and cultural repertoires/cleavage structures/discursive opportunities as strongly connected social entities. Without repertoires, cleavages, and discourses, people simply cannot make sense of life, and events cannot occur. Yet given the ruptures that arise in response to events, it is also important to consider scenarios where they are catalysts for a change in people's behavior and attitudes. I introduce a typology that helps to understand why some happenings do not become events (which means that the existing repertoires, cleavages, and discourses remain the same), whereas others do (which implies transformation). This typology indicates that happenings either turn into events because they are significant confirmations of existing cultural or political patterns (“focus events”) or because they significantly break with them (“shock events”).

In the second part of the paper, then, I show how this typology can be used to make sense of a concrete event‐case: the distinctive framings of 9/11 in the American and Dutch public spheres. In prior research (van Dooremalen & Uitermark, 2020), I have found that within American debates over domestic affairs, this event has been dominantly related to the issue of safety, whereas in the Netherlands, this has been the case for the issue of Islam. The event typology helps to explain this variation, as demonstrated by a follow‐up investigation analyzing how and why public actors in the two countries have drawn links between these issues and 9/11 in national newspapers and legislative debates.

## 2 EVENTS IN CULTURAL REPERTOIRES, CLEAVAGE STRUCTURES, AND DISCURSIVE OPPORTUNITIES

The three perspectives I review differ substantially with respect to the types of behavior and attitudes they aim to analyze. Lamont's work is cultural‐sociological. In various books, she and her collaborators show that the “symbolic boundaries”—conceptions of good and bad, who belongs and who does not—drawn in the national contexts of the United States and France are vastly different: among upper middle‐class men (Lamont, 1992); among working‐class men (Lamont, 2000); and in various fields and settings, such as debates over racism, journalistic habits, and the Rotary Club (Lamont & Thévenot, 2000). They explain these variations in particular by using the concept of “cultural repertories”: the sets of dominant cultural notions that exist in given national contexts.

Kriesi's concept of “cleavage structures” attempts to understand the hierarchy of “cleavages”—religious, economic, or socio‐cultural chasms—in national political contexts. By using this concept, Kriesi et al., (1995) explain the different degrees of success that new social movements have enjoyed in Western European countries. The perspective has also been reversed, to investigate whether globalization processes (Kriesi et al., 2008), and the recent economic crisis (Kriesi & Pappas, 2015) has led to a convergence or divergence of cleavage structures across countries.

The work of Koopmans likewise addresses the structural aspects of national political landscapes. However, he is less concerned with the saliency of national debates, instead focusing on specific discursive notions: what ideas are socially (un)acceptable and how does this affect social life? Using the concept of “discursive opportunity structures,” Koopmans and his collaborators reveal how different forms and levels of media attention to radical right‐wing violence in German regions influence its nature and scope (Koopmans & Olzak, 2004), as well as the extent to which ideas of cultural and ethnic citizenship vary between Western European countries (Koopmans et al., 2005).

Apart from the different types of behavior and attitudes they aim to understand, what all three of these perspectives have in common is the social constructivist vision that cross‐national differences in the prominence of certain discussions or ideas are not determined by variations in objective factors like population size or the economic situation *as such* (Lamont, 1992, pp. 134–136; Kriesi et al., 1995, pp. 145–164; Ushiyama, 2019, p. 1,736). Instead, the importance of such factors is always mediated by the meanings attached to them within given repertoires, cleavages, and discourses. For instance, following Kriesi's notion of cleavages, we could argue that population size becomes more significant in a country's political discussions if demographic issues increase in saliency.

Notwithstanding the important contributions these perspectives have made to understanding cultural and political behavior and attitudes, what is missing from each is a thorough answer to the question of their relation to events. Indeed, events are incorporated at different points in the aforementioned books and articles. Yet, the theoretical problem is that this is mostly done in such a way that it appears to be obvious a priori how events “work.” Consequently, they become “black boxes”—social entities whose ontological statuses are assumed to be clear, without further explanation. Sometimes, they are presented as an outcome of existing “interpretative structures” (a term that I use from this point on to refer to cultural repertoires, cleavage structures, and discursive opportunities, taken together). This is the case, for example, in Koopmans and Olzak (2004) about the relationship between media attention and radical right‐wing violence in Germany as well as in Lamont et al. (2016) regarding responses to stigmatization among marginalized groups in the United States, Brazil, and Israel. Both works suggest that discursive opportunities or cultural repertoires “color” the framing of a certain happening, or even cause it.

This is how social life often takes shape. Yet, there is also the possibility that certain happenings are perceived as being so remarkable that they are not only colored by existing interpretative structures, but a situation arises in which structures themselves are transformed. This is where the notion of the event becomes relevant.

Although these three perspectives purport that social life is more continuous than mutable, scholars who use them at some points in their work refer to the possibility of transformation in response to events. For instance, Lamont (1992) claims that the French Revolution has been an important factor in the creation of French cultural repertoires:

The French equivalent of “Americanism” can be found in the Republican ideals of the French Revolution, which still survive today. These ideals include the Jacobin obsession with equality, universalism, and national unity that negates particularism based on locality, corporate membership, and birth (Lamont, 1992, pp. 137–138).

Yet, although this claim seems plausible, Lamont's book does not give proof that this causal link indeed exists. The approach of Kriesi and Pappas (2015) to the recent economic crisis offers more in this regard. It analyzes how the crisis has had varying effects on the success of populist political parties in European countries. However, although Kriesi and Pappas (2015, p. 7) take this event as a starting point, they do not conceptualize exactly how it “works.” The crisis is defined as a series of points in time after which there either has or has not been an effect. Their book lacks an explanation of the circumstances under which economic crises lead to political change. Thus, the missing link in each of the three theoretical perspectives is an understanding of how a happening can become an incentive to transform the existing interpretative structures.

## 3 BRINGING EVENTS IN: A TYPOLOGY

To make this link, I propose not examining interpretative structures and events as isolated phenomena in which either structure determines event (Koopmans & Olzak, 2004; Lamont et al., 2016) or event determines structure (Lamont, 1992; Kriesi & Pappas, 2015). Instead, I argue that structure and event should be conceptualized as strongly linked to one another. Without interpretative structures, it is impossible to attach meanings to an event. For instance, an economic crisis is only considered a crisis within a context of shared assumptions about economic adversity. However, it is also difficult to imagine that the disruptive power of events will never compel people to change the interpretative structures by which they live. Following Sewell (2005, pp. 226–228), we can conceptualize social life as a sequence of happenings. Every day, people encounter many happenings, and in most cases their lives do not alter that much. This is because most of them are simply too ordinary to invoke a transformative response. Sewell's central theoretical claim concerning events is that they are exceptional happenings: they bring people into conflict with their own worldviews, which they consider changing as a result.

The question is then: when do happenings become events? This must have something to do with the happening's relation to existing interpretative structures. Event literature offers different views of this relationship. Some scholars suggest that events occur when a happening is *in contradiction* with dominant interpretative structures. Sewell (2005) argues this in his analysis of the storming of the Bastille, as does Sahlins (1985) regarding Hawaiians’ misidentification of Captain Cook as their God Lono. Both scholars consider the happening's shock effect central. Because it was so unexpected that ordinary citizens would start a revolution in 1789 in France or Hawaiians were confronted with the appearance of one of their gods these happenings became events.

Other event scholars pay less attention to the relationship between happenings and interpretative structures. For instance, Swidler (1986, pp. 278–280), Wagner‐Pacifici (2017), and Zolberg (1972, pp. 183–184) consider the temporality of events—the point in time at which they take place or their duration—more important.

Yet, in Kingdon's [2011 (1984)] book on policy processes it is key. He claims that happenings do not become important (eventful) because they are *in contradiction* with existing central debates or discursive notions, as Sewell (2005) and Sahlins (1985) suggest, but rather since they are *in consonance* with them. He develops the idea of “focus events” to describe this kind of happenings and uses the American policy agenda during the 1970s as his case study. A prominent focus event in this period was the collapse of Penn Central railroad station in 1970. This incident became a symbol for problems in the American transportation system, which had already been under discussion for some time (Kingdon, 2011 [1984], p. 96). Kingdon formulates the character of focus events thus^1^:

In general, such a symbol acts (much as personal experiences) as reinforcement for something already taking place and as something that rather powerfully focuses attention, rather than as a prime mover in agenda setting. Symbols catch on and have important focusing effects because they capture in a nutshell some sort of reality that people already sense in a vaguer, more diffuse way (Kingdon, 2011 [1984], pp. 97–98).

​

So, in event literature, we find two notions—“shock events” and “focus events”—which resemble opposing views on the relationship between events and interpretative structures. In the case of a shock event, a break with the existing dominant interpretative structures occurs. Consequently, observers often describe this type of event using grand characterizations, such as “this is totally unexpected” or “the world will never be the same again.” Conversely, in the case of a focus event, the happening confirms dominant interpretative structures and is thus usually characterized by phrases such as “we already knew that…” or “this happening proves that...” Because of the stronger emphasis on continuities, the resulting changes of focus events will frequently be smaller compared with the responses to shock events—the radicalization of dominant discursive notions (focus) versus the sudden emergence of new ideas (shock).

I propose that the three distinct kinds of occurrences I have discussed—ordinary happenings, shock events, and focus events—can be integrated into a typology regarding the relationship between happenings and interpretative structures. This is illustrated in Table 1.

**TABLE 1:** A typology of the relationship between different types of happenings and interpretative structures

		Extent of transformation	
		No transformation	Transformation
Extent of surprise	*No surprise*	Ordinary	Focus event
		Happening	
	*Surprise*	Inconceivable	Shock event
		Happening	

The vertical axis shows the degree to which the happening is unexpected or surprising. The horizontal axis indicates the extent to which it provides a motive for transformation. An ordinary happening is an occurrence so common that it is not remarkable enough to become an event (Sewell, 2005, pp. 226–228). A focus event emerges from confirmation, whereas a shock event is caused by surprise. Finally, there is a type of happening that does not incite transformations, even though it is surprising. These include occasions that have the potential to shock, but for which there exists no vocabulary to turn them into events, or for which there are political or moral obligations not to do so. An example of the former would be a “family drama,” a person killing family members. In many Western countries, the media treat such happenings with shock, but they generally do not link them to a larger societal problem (or in the words of Alexander (2018): these occurrences are not “societalized”). An example of the latter is Western communists’ historic refusal to acknowledge the existence of the Soviet Gulag, to preserve the legitimacy of their political project. I call these kinds of happenings “inconceivable happenings.”

The happenings in the typology are—obviously—very much ideal types. In empirical reality, many occurrences will have in‐between positions somewhere at the two axes. In addition, it is important to emphasize that a happening's position within the typology can potentially change over time (*cf*. Wagner‐Pacifici, 2017), as Alexander (2002) demonstrates with the Holocaust. When news first broke in 1942 of the mass extermination of European Jewry, the Holocaust was “coded” as yet another example of Nazi immorality—a focus event. Over the course of the decades that followed, coding of the Holocaust transformed into its position as the dominant representation of absolute evil—a shock event.

## 4 CASES, DATA, AND METHODS

### 4.1 Case selection: 9/11 in the American and Dutch public spheres

In the remainder of this paper, I illustrate the analytical usefulness of the typology, by researching responses to a concrete event case. An ideal illustrative case would be an occurrence, which has attained significantly different cross‐national meanings. Delving into contrasts can show how event effects are produced through the specific relationships between a happening and national interpretative structure [*cf*. Della Porta (2008, p. 216) on most‐different system designs].

The framing of 9/11 in the American and Dutch public spheres offers such a case. In prior research, I have found that significantly different meanings were attached to this event in national newspapers from the two countries over the 2001–2015 period (van Dooremalen & Uitermark, 2020). An important part of this research consisted of a topic‐modeling investigation. This is an automated content analysis technique that produces rows of words—topics—that often appear together in large text corpora (Blei, 2012; Grimmer & Stewart, 2013). I used it to find out which meanings were related to 9/11 within the national press of the two countries.^2^ For each year I determined the 20 most prominent topics in national newspaper articles, which included the search term “September 11” (or “11 September” in Dutch).^3^ This resulted in lists of hundreds of topics for both countries. To facilitate a comparison, another researcher and I independently of one another gave issue codes to these topics (*cf*. DiMaggio et al., 2013; Marshall, 2013). A significant number of codes concerned foreign issues such as the War in Afghanistan and the Iraq War, as well as domestic issues like safety, Muslim integration, or national politics (see van Dooremalen and Uitermark (2020) for a more extensive explanation of the entire analysis).

**GRAPH 1:**
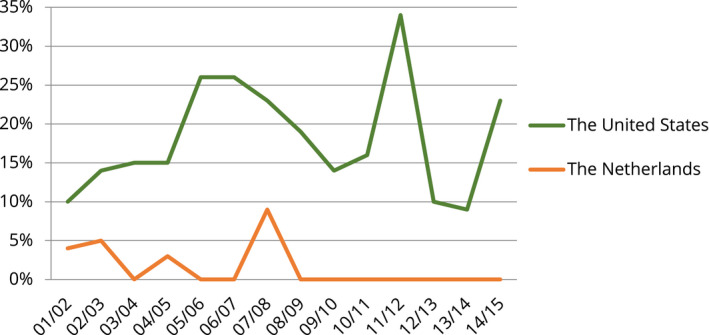
Yearly Percentages of American and Dutch Newspaper Articles Presenting 9/11 as a Domestic Safety Event

**GRAPH 2:**
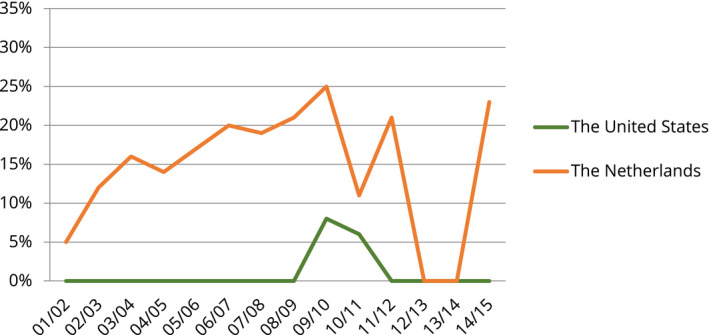
Yearly Percentages of American and Dutch Newspaper Articles Presenting 9/11 as a Domestic Islam Event

This investigation's most important result for the purpose of this paper is that within American and Dutch national newspapers 9/11 was understood quite differently as it related to domestic issues. In American newspapers, the most prominent domestic issue connected with the event was safety. Many topics refer to subjects such as surveillance in buildings within the United States and preventing terrorist attacks on airplanes or trains on American soil. In Dutch newspapers, on the other hand, the central domestic issue of contention was the integration of Muslims into Dutch society. 9/11 was linked to a plurality of topics dealing with the position of Islam in the Netherlands. Graph 1 and 2 give an overview of these results. They illustrate the relative sizes of the topics (measured as percentages) in American and Dutch newspaper content about 9/11, which address it as a safety and Islam event, respectively. What is remarkable is not only the fact that the central domestic issues of contention differ but also that both issues are seldom among the top 20 topics in the other country during most years of analysis.

### 4.2 Illustrating the typology: An in‐depth analysis of newspapers and legislative debates

How can we, using the developed event typology, understand these cross‐nationally diverse meanings of the same event? I answer this question by conducting a follow‐up analysis of the aforementioned research. This analysis concerns two data sources from both countries: national newspaper articles and debates in national legislatures. Newspapers and legislatures are two central arenas within contemporary public spheres (*cf*. Koopmans & Statham, 2010)—social spaces where public elites give their opinions about societal matters and that influence the opinions of the general public.

My investigation mainly focuses on the first month after 9/11. This gives an overview of the initial phase in which the associations between the event and the two issues are created. However, one of the remarkable things I found in my prior research is that these associations were very consistent over time (Graphs 1 and 2). To further delve into this issue, I also analyze associations made between 9/11 and the two issues during the first weeks following the most important domestic terrorist attack committed by Muslim extremists in each country. In the Netherlands, this is the murder of filmmaker Theo van Gogh, in November 2004, and in the United States, it is the attack on the gay night club in Orlando, in June 2016. Even though these two attacks had specific targets, they, like 9/11, can very well be considered national events. In both cases, the terrorist himself stated—via a note on Van Gogh's body and a 9–1–1 call—that his attack was (also) an assault on the Netherlands and the United States. More important, research indicates that both occurrences have been discussed in public debate as events with nationwide implications (Buruma, 2006; Elmasry & el‐Nawawy, 2020; Eyerman, 2008; Haider, 2016).

The newspaper articles for my analysis come from the same sources that I used for the analysis outlined above (see footnote 2). The debates I accessed through the American website congress.gov and the Dutch officielebekendmakingen.nl. I searched for articles and debates with references to 9/11 (which I measure again with “September 11” and “11 September”) and terms related to the two domestic issues (“Islam,” “Muslims,” “safety,” “security”). For the two domestic attack cases, I added, respectively, “van Gogh” and “Orlando” as search terms. In total, I analyzed 1,893 newspaper articles and 21 legislative debates from the United States, and 532 articles and 14 debates from the Netherlands.

Because my goal is to illustrate the event typology, I aim to give rich, in‐depth descriptions of the data (*cf*. footnote 4 in Alexander (2018, p. 1071)). Therefore, I explore them by engaging in a qualitative content analysis (*cf*. Kohlbacher, 2006; Kuckartz, 2014). Having the typology in mind, I try to answer the following questions. How is 9/11 framed with regard to safety and Islam? Is it interpreted as a significant happening (an event)? If so, is this because it is a confirmation of debates that were already going on (focus), or since it is in opposition to them (shock)? And if 9/11 is not seen as an event, why is that so? Is it because it is simply not considered significant enough (an ordinary happening) or because there are reasons that “block” its transformation into an event (an inconceivable happening)?

Following the shared assumption of the three theoretical perspectives (Lamont, 1992, pp. 134–136; Kriesi et al., 1995, pp. 145–164; Ushiyama, 2019, p. 1736), my aim is not to find out how objective factors such as country size or the locus of the attacks of 9/11 can directly explain the event's meaning‐making processes. Rather, my goal is to take the perspective of public actors themselves and investigate which factors and justifications they mobilize, while drawing on national interpretative structures, to claim 9/11 to be (not so) shocking or to link it to the issues of safety and Islam. For instance, if the locus of the attacks is frequently mentioned by these actors, this is a sign that this factor, through mediation of the interpretative structures, indirectly plays a role for their event framings. I convey data by using citation representative of various framings of the event, with a specific focus on those public actors who possess the “discursive power” (Koopmans & Olzak, 2004; Uitermark, 2012) to significantly influence existing interpretative structures. Obvious examples of such actors are the U.S. president or the Dutch prime minister.

## 5 9/11 IN THE AMERICAN PUBLIC SPHERE

### 5.1 A shocking safety event

Why is it that 9/11 has been so strongly associated with the issue of safety in the American public sphere? This is an obvious case of a shock event. Many American public actors depict 9/11 as an occurrence, which breaks firmly with existing cultural repertoires of national safety. Immediately after the attacks, it is evident to nearly all of them that radical policy shifts regarding this issue must be implemented. Newspaper articles from the days following 9/11 use a wide variety of typifications, all of which indicate that the experience surpasses the scope of possible scenarios imagined by Americans. An article in *The Washington Post* from the day after the attacks (12 September) states, for example:

The nightmare scenario of the post‐Cold War era—terrorism at home aimed at innocent civilians—hit with a terrible swiftness and frightening power that even the experts had not imagined, and it is clear that Sept. 11 has become the defining day of Bush’s presidency.

The fact that “even” experts did not expect the attacks elevate the element of shock. What is interesting about many of the political analyses from the first days following the event is that they take the long‐term history of terrorism in the world into consideration, but nonetheless emphasize that even with this knowledge in mind the occurrence of 9/11 is still very surprising.

For example, Eleanor Holmes Norton, a Democratic delegate to the House of Representatives, declared at a Congress meeting on 11 September that these attacks “transcended” all existing discussions about national safety:

This shows us that whatever we were doing should be history, and that we need to start all over again. We are much too late in recognizing that the nature of war has changed dramatically .… Today, we looked like we were operating in the 19^th^ century.

In other words, the logic of confirming or negating expectations is used to amplify the enormity of the event—Americans could imagine many bad scenarios, but this one nevertheless exceeded all realms of possibility.

The fact that in the United States 9/11 is a shocking event for cultural repertoires regarding domestic safety may also be derived from the almost unanimous call by American public actors for a strong political response. Even those critical of the introduction of stricter security measures see the attacks as an inevitable motive for implementing radically new policies. They may be skeptical of and unwelcoming to such actions, but this occurrence is so big and shocking that they consider maintaining their existing discursive rules no longer “legitimate” (*cf*. Koopmans & Olzak, 2004, pp. 204–206). An editorial commentary published in *The* *New York Times* proposed, for example (12 September 2001):

Inevitably, the attacks will make daily life in the United States more complicated. Security will be tightened at private buildings and federal offices. Airport checks will be stricter and more frequent, requiring passengers to arrive earlier. In general, it will be harder to get about.

What are the reasons for this immense shock to American public actors’ domestic safety repertoires? Among the various justifications given, two stand out. The most important is the sudden realization that the United States is not as militarily impenetrable as it had long considered itself to be. This change in discourse is, for instance, put forward by *The* *New York Times* columnist Michael Gordon (12 September 2001): “Nobody doubts America's clear military superiority. But the lesson of yesterday seemed to be that even such power is vulnerable and may offer no effective redress against terror.” Another reason for the shock is the realization that the United States had not been attacked since World War II and for an even more substantial period not on its own territory. This had long turned domestic safety affairs into a relatively “nonsalient topic” within its political cleavage structure (*cf*. Kriesi et al., 2008, pp. 9–14). Accordingly, *Daily News* staff writers Heidi Evans and Thomas Hackett claim (12 September 2001): “It's a nationwide anxiety that people elsewhere in the world have long experienced, but not here.”

These two elements of the interpretative structures that are being challenged come together in the omnipresent comparison to the December 7, 1941 Attack on Pearl Harbor. In the first week after 9/11, only the comparison with the assassination of President John F. Kennedy is made more frequently in the materials I analyzed (375 vs. 428 hits, out of 2,842 articles). The Pearl Harbor Attack also came unexpected and similarly undermined American feelings of military superiority. In addition, the fact that Pearl Harbor happened six decades prior to 2001 indicates for many American public actors how relatively safe their country has been during the years in between.

### 5.2 An inconceivable Islam happening

Next, why is there no clear link in the United States between 9/11 and its Muslim citizens? Although several reasons explain the framing of the event as a domestic safety issue, only one clear reason exists why it is not interpreted as a domestic Islam issue: the dominant cultural repertoire around 2001 demands that Americans do not draw “moral boundaries” (Lamont, 1992, p. 4) with Muslim citizens on the basis of their religion. This makes it unacceptable for politicians and commentators to problematize the societal status of American Muslims in response to 9/11 and thus to link issue and event.

When there are discussions about the danger of Islam among American political and cultural elites in the weeks following the event, this is mostly presented as an external problem rather than an internal, domestic affair. Perhaps Muslims living in Afghanistan and Saudi Arabia are dangerous, but not those living in the United States (Alba & Foner, 2015, pp. 125 – 130). Indeed, during the direct aftermath of 9/11, there was a backlash in everyday life against American Muslims (Bakalian & Bezorgmehr, 2009; Cainkar, 2009). For instance, a substantial number of mosques were attacked. However, among American public actors, there is no acceptance for this type of behavior—it is generally condemned. It is likewise considered discursively illegitimate to ask American Muslims to “take a stance” against Islamic terrorism.

The actions of President George W. Bush in the weeks following the attacks exemplify the larger American discourse regarding this issue. A few days after the event, he visits the Islamic Center in Washington and declares that “Islam is peace.” In *The Washington Post* (September 14, 2001), his stance is quoted thus: “We should not hold one who is Muslim responsible for an act of terror”. Beyond this, in his first speech to Congress after 9/11 (September 20, 2001), he has a special message for American Muslims:

We respect your faith. It is practiced freely by many millions of Americans, and by millions more in countries that America counts as friends. Its teachings are good and peaceful, and those who commit evil in the name of Allah blaspheme the name of Allah.

What is remarkable is that this stance receives “supportive resonance” (“consonance”) from actors of all political stripes—a key characteristic of a powerful discourse (Koopmans & Olzak, 2004, pp. 204–205). Journalist Walter Shapiro, a liberal, for instance, writes in a column (*USA Today*, September 14, 2001): “President Bush should be hailed for his unequivocal efforts to prevent ethnic and religious scapegoating.” The historical references used in these expressions of appreciation allude to episodes in American history when ethnic minorities were scapegoated—for example, Japanese Americans after Pearl Harbor in 1941 and American Muslims following the first attack on the World Trade Center in 1993—and those actions are now generally strongly condemned.

In the American public sphere, 9/11, thus, appears to be an “inconceivable happening” with regard to the issue of Islam. The happening is shocking for American public actors, but it is unthinkable for them to draw the conclusion from it that there is something wrong with Muslims living in the United States. To entertain such an idea is generally considered discursively illegitimate and un‐American (i.e., incongruous with the repertoire of avoiding public criticism of Muslim citizens on the basis of their religion).

This analysis contrasts the claim that in the early post‐9/11 context the discursive opportunities to stigmatize American Muslims had widened (Garg et al., 2018; Woods & Arthur, 2014). It likewise confirms investigations of scholars indicating that the attacks did not result in such a discursive shift, as American public actors framed this group positively during the event's initial aftermath (Bail, 2014; Ibrahim, 2010, pp. 117–119).

It should be noted, although, that in tandem with these *explicit* pro‐Muslim messages, antiterrorism policies were developed that increased the *implicit* stigmatization of American Muslims. These measures often resulted in an administrative focus on specific groups, such as people with a “terrorist” (meaning: Middle Eastern) look (Byng, 2008). Another, and possibly more noteworthy, observation is that although my prior research indicated no link between 9/11 and Islam as a domestic issue in the years following the event (see Graph 2), Christopher Bail's book (2014), in contrast, shows that anti‐Muslim messages actually gained “visibility” and “consonance” (*cf*. Koopmans & Olzak, 2004, pp. 203–206) during this period. The question, although, is whether 9/11 played a significant role in this discursive development.

My analysis of the Orlando shootings in June 2016 suggests that this is not the case. Indeed, in the first days following that tragedy—in contrast to the period following 9/11—various politicians and commentators claim that this attack points to a larger “Islam problem.” The most prominent examples are Donald Trump (by then a presidential candidate), who proposes a ban on Muslims entering the United States, and Senator Marco Rubio, also a presidential candidate, who states that this attack does have a religious component, thus breaching the former cultural repertoire of nonjudging: “Common sense tells you he specifically targeted the gay community because of the views that exist in the radical Islamic community with regard to the gay community” (*The* *Washington Post*, June 13, 2016). It is mostly right‐wing politicians and commentators who use this discourse. President Barack Obama and presidential candidate Hillary Clinton, for example, hold on to the old repertoire. They are reluctant to blame the religion of Islam, or Islamic worldviews, and claim that doing so is “un‐American and offensive” (*The* *New York Times*, June 16, 2016).

9/11, however, is hardly mentioned in the anti‐Muslim discourses. Donald Trump is actually one of the few public actors who refers to the event in these discourses, stating that it revealed problematic aspects of Islam, because—he claims—Muslims in New Jersey were cheering in response to it (*The Washington Post*, June 22, 2016). This discourse receives little or no consonance from other actors. When they refer to 9/11, it is (still) only as the biggest terrorist attack in American history and as a shocking safety event. Thus, I find that in the United States, up until the Summer of 2016, the occurrence was generally considered an inconceivable happening regarding Muslim integration.

The results of the analysis of the links between 9/11 and the two domestic issues in the American public sphere are summarized in Table 2.

**TABLE 2:** 9/11 and safety and Islam as domestic issues in the United States

		Extent of transformation	
		No transformation	Transformation
Extent of surprise	*No surprise*		
	*Surprise*	Islam (Inconceivable happening)	Safety (Shock event)

## 6 9/11 IN THE DUTCH PUBLIC SPHERE

### 6.1 A focusing Islam event

The Dutch public discussion about Muslim immigrants after 9/11 is completely different from the American one. A cultural repertoire that problematizes the relationship between Muslim immigrants and Dutch society is widely present. Not only right‐wing public actors but also leftists express this discourse. For example, left‐wing columnist Jan Blokker comments sarcastically that several days before 9/11, the Dutch Islamic Broadcast Society (NMO) aired a video featuring cruel quotes from the Quran about non‐believers (*De* *Volkskrant*, September 15, 2001):

Even though Lower Manhattan had not yet been destroyed, and 5,000 to 10,000 more Americans were alive than two days later, the September 11^th^ hijackers had already bought their plane tickets, and they undoubtedly felt strengthened in their resolve to commit their murders by the fact that the NMO was transmitting this message not less than five times a day.^4^

During the first week after 9/11, a lot of discussion about Samuel Huntington's “clash of civilizations”‐thesis ensues. The event is generally interpreted as confirmation of his idea that Islam has a problematic relationship with the Western world. Even though he is an American scholar, I found 15 references to Huntington in a total of 64 Dutch Islam–related newspaper articles published during the first week after the attacks, whereas I only came across one such reference among 299 articles from U.S. newspapers for the same period.

Later, many Dutch public actors believe Huntington's thesis to have been further confirmed, when surveys indicate that substantial numbers of Dutch Muslims say that they respect the terrorists, and it is reported that a significant amount of Muslim children did not want to remain silent during 9/11 commemoration ceremonies in schools. Right‐wing commentator Sylvain Ephimenco is very critical of this behavior. He states in “An Open Letter to Muslims in the Netherlands” (*Trouw*, September 29, 2001):

How salutary it would have been—this is only a personal wish—if, in order to narrow the divide, shortly after the attacks, Muslims and non‐Muslims had come together in the streets to express their aversion to terrorism. Evidently, it is never too late. Neither is it too late [for Muslims, article’s author] to abandon the familiar stands of victimhood, so that the prejudices of non‐Muslims can be debunked.

The existence of this type of opinion reveals a significant difference between the American and Dutch discursive opportunities concerning this issue: whereas castigating native Muslims citizens for their responses to the attacks is nearly absent from the American early post‐9/11 context, it is highly visible and considered legitimate in the Dutch one.

At the same time, the astonishing terms used by American public actors, which turn 9/11 into a shocking safety event, are not found in Dutch associations of the event to Islam. Indeed, the attacks themselves are represented as a shock. However, that *Muslim* terrorists are responsible does not cause much surprise. What is striking, when analyzing the links drawn between 9/11 and Dutch Muslims in the first month following the event, is that they reveal indications that critical repertoires and discourses about this group had already been “in place” for at least several years. For example, journalist Dirk Vlasblom writes two days after the attacks (*NRC* *Handelsblad*, September 13, 2001): “Since the end of the Cold War, the Western world has a new enemy: Islam in its fundamentalist form”. Another article (*De* *Volkskrant*, September 15, 2001) makes a clear link to Dutch public discussions in the Netherlands after the Salman Rushdie affair:

They [a Turkish‐Dutch organization, article’s author] distributed a press release stating that an attack on the West is also an attack on the Western freedom of religion, and therefore, in fact, also on Islam itself. This is a big improvement compared to 1988, the year in which the British‐Indian writer Salman Rushdie was subject to a fatwa by the Ayatollahs for his book, *The Satanic Verses*. At that time, Muslim intellectuals were more reticent.

A different reference often made is to the heated discussions about the Dutch Imam El Moumni, who in spring 2001 called homosexuality “a disease.” If we consider the discourses regarding this incident used by two of the commentators quoted above, then it seems that the cultural repertoire to problematize the societal position of Islam did not arise suddenly after September 11, 2001. Jan Blokker announced his fear—again sarcastically—that El Moumni's ideas would gain wider acceptance (*De* *Volkskrant*, May 12, 2001): “Soon no homosexual will dare walk around in his thong when the weather is nice, out of fear of religious Turks or Moroccans, who have been indoctrinated by their mosques to wage jihad.” Sylvain Ephimenco wrote an “open letter” (as he did after 9/11) to El Moumni in the opinion magazine *De* *Groene Amsterdammer* (9 June 2001).^5^ He ended it with a reaction to another imam, who had claimed that Muslims ought not to be friends with non‐Muslims: “Dear Imam, if this really is a generally accepted view by you and your colleagues, then my opinion is correct: Islam is a disease which should be cured over time.”

In sum, three elements are central to the association of 9/11 with Dutch Muslims: (1) there is a widely visible tendency to problematize the relationship between Islam and Dutch society, even among leftist public actors; (2) there is little shock, but instead a strong continuity such that 9/11 is related to Dutch political discussions about Islam already taking place; and (3) together, these elements make it natural for public actors to draw a strong link between issue and event (Graph 2).

These findings go against the idea that the occurrence was the main discursive turning point, a shock event, in Dutch debates about Muslim immigrants—an idea which is rather dominant in this literature (Buijs, 2009, p. 422; Roggeband & Vliegenthart, 2007, pp. 306–315; Tillie, 2008, pp. 19–21). Instead, they undergird work that claims that the development of these debates has been much more gradual and that the public problematization of the relationship between Islam and Dutch society had already started during the 1980s and 1990s (for instance, Uitermark, 2012; van Reekum, 2016). Thus, my analysis indicates that 9/11 was a focus event regarding the issue of Islam; it has intensified the “salience” of the immigration/integration‐cleavage within the Dutch public sphere (Kriesi et al., 2008, pp 154–182).

### 6.2 An inconceivable safety happening

And what about Dutch responses to 9/11 concerning the issue of safety? Those are actually expressed in more shocking terms than those made regarding the issue of Islam. Although the initial reactions in the Netherlands are framed in less alarming terms than in the United States, indications of surprise and shock are widely visible. For instance, one day after the event, Prime Minister Wim Kok (PvdA, Social Democrats) states in parliament on behalf of the Dutch government: “The indescribable catastrophe which has hit the American people, fills us with bewilderment and horror.” In addition, in her yearly speech from the throne, which took place a week after 9/11, Queen Beatrix says that the attacks “remind us of the fragility of our existence” (*NRC* *Handelsblad*, September 18, 2001). Security expert Uri Rosenthal writes in an opinion piece (*De* *Volkskrant*, September 18, 2001):

The events were literally unthinkable: they embodied a combination of at least three terrorist acts; an attack on the icons of what was once called the military‐industrial complex; an acute threat of a prospect of more similar attacks; and an incomprehensible number of deaths in New York.

Thus, during the first week after 9/11, it seems that likewise in the Dutch public sphere the event is going to be associated with a loss of safety—both nationally and internationally. However, in subsequent weeks, it is rarely linked to Dutch national safety concerns. No domestic safety policies are proposed in response to it.

This is basically also the case after the Van Gogh murder, in November 2004. During the first month following this event, 25 articles were published in Dutch national newspapers that contain the term “safety” (along with “Van Gogh” and “9/11”). Yet in most of these articles—around 15 of them—general policy proposals to increase safety were hardly visible, but concerned the specific issue of Islamic terrorism. For instance, they included suggestions to hold more public debates that involve Dutch Muslims and non‐Muslims or to intensify the proliferation of “de‐radicalization programs” for Muslim extremists. So, the combination of 9/11 and the Van Gogh murder is mostly interpreted as two “Islam events,” rather than as two “safety events”—even within newspaper articles that contain the term “safety.” In those articles that relate the events to broader safety measures, those are mostly framed as an international rather than a national Dutch issue. For instance, an article discussing a policy report, which came out 2 weeks after the Van Gogh murder (*NRC* *Handelsblad*, November 17, 2004) claims:

Since the 11 September 2001 attacks, the lack of international cooperation between intelligence and security services has been considered one of the biggest obstacles in the fight against international terrorism. Security services stick to national borders, whereas terrorist networks are characterized by the fact that they can no longer be linked to one, two, or even ten countries.

The reason for the lack of substantial association between 9/11 and domestic safety issues is never made explicit; implicitly, however, Dutch public actors mobilize various justifying cultural repertoires, of which two are dominant. Both indicate that regarding the issue of safety, 9/11 has become an “inconceivable happening” in the Dutch public sphere. The first one occurs in the above citation: the Netherlands is considered simply too small a country for a big problem like terrorism. Dutch public actors adhere to the “repertoire of evaluation” (*cf*. Lamont & Thévenot, 2000, pp. 4–8) that a topic of such immense scale needs to be tackled at an international level.

Furthermore, changes in security policies are viewed with greater ambivalence in the Netherlands than in the United States. Many of its public actors question both their effectiveness and moral justness. This ambivalent cultural repertoire is demonstrated in the following quote from an opinion article by technology lawyer Anne‐Marie Kemna (*De* *Volkskrant*, October 2, 2001):

Apart from the discussion regarding civil rights, it is also highly questionable whether terrorist and criminal organizations can be detected with large‐scale detection practices, the introduction of an ID requirement, and restricting encryption. Is this the right way to target such groups?

A summary of the analyses of the associations between 9/11 and the domestic issues of Islam and safety in the Dutch public sphere is provided in Table 3.

**TABLE 3:** 9/11 and safety and Islam as domestic issues in the Netherlands

		Extent of transformation	
		No transformation	Transformation
Extent of surprise	*No surprise*		Islam (Focus event)
	*Surprise*	Safety (Inconceivable happening)	

## 7 CONCLUSIONS

The central aim of this paper is to better understand how happenings (do not) turn into events. I propose that this can be done not by conceptualizing events and interpretative structures—“cultural repertoires” (e.g., Lamont, 1992, 2000, 2019; Lamont et al., 2016; Lamont & Thévenot, 2000), “cleavage structures” (e.g., Kriesi et al., 1995, 2008; Kriesi & Pappas, 2015), and “discursive opportunity structures” (e.g., Koopmans & Olzak, 2004; Koopmans & Statham, 2010; Koopmans et al., 2005; Ushiyama, 2019)—as separate social entities, but rather by developing a typology that indicates why some happenings are “just” reproductions of said structures, whereas others are so significant that they become incentives to transform those very structures. This typology posits that such a transformation can occur either through shock (Sewell, 2005) or focus (Kingdon, 2011 [1984]): happenings turn into events because they are a significant rejection or since they present a significant confirmation of the ideas and expectations that comprise existing interpretative structures.

The analysis of the case of 9/11 in the American and Dutch public spheres then illustrated that this typology can be applied empirically by analyzing how public actors link happenings and interpretative structures. If they react surprised, a happening is a shock event (in this analysis, safety in the U.S.). When they indicate its significance, while at the same time emphasizing the fit with their expectations, it is a focus event (Islam in the Netherlands). In cases where they would mention the happening's significance, without inferring consequences, it will be an inconceivable happening (both Islam in the United States and safety in the Netherlands). And when there is hardly any mention of its significance or no reference to it at all, the occurrence is an ordinary happening (no cases in this analysis). These are rather absolute research outcomes, given the approach that I introduced in Section 3, which assumed gradual differences between the four ideal typical happenings. This might be a product of choosing 9/11 as a case. Analyzing an event of less significance than one of the biggest occurrences of the last decades would probably lead to less extreme findings.

Because I aimed to offer a rich, in‐depth illustration of the typology, I used qualitative content analysis to explore my data. An obvious limitation of such an approach is the subjectivity of the analyst, creating possible biases in the findings (e.g., cherry‐picking quotes). However, the typology can also be used for doing forms of text analysis that are generally considered more objective, such as manual quantitative content analysis (e.g., a claim making investigation of the number of instances of shock or focus in event reactions (*cf*. Koopmans et al., 2005)) or even automated text analysis (e.g., using a dictionary approach or supervised learning methods (Grimmer & Stewart, 2013:274—280) to measure extents of shock, focus, inconceivability, or ordinality).

As a final note, it is interesting to reflect on the conditions for change of a happening's position in the typology. My findings indicate that in the United States, up until the Summer of 2016 (the Orlando shooting), 9/11 was (still) not viewed as an event regarding Muslim integration—even though anti‐Muslim public discourses had become widespread by then. Why? It may be that 9/11 has become “locked in”—to use the terminology of path dependency scholars (e.g., Mahoney, 2000)—as an American inconceivable happening concerning Muslim integration. This would imply that once a certain meaning is attached to an event, this initial framing does not shift so easily. Future research could delve into the persistence of such event “lock‐in effects.”

In contrast, the opposite perspective is also interesting to analyze: what is required for a happening's initial framing to be changed? A turn in public discourses about American Muslims along with the occurrence of the Orlando shooting apparently were not enough to create a “code switch” (Alexander, 2002) of 9/11’s central meanings. What would probably be needed are social movements or actors with a lot of discursive power (*cf*. Koopmans & Olzak, 2004; Uitermark, 2012) who would specifically pursue such a transformation of the event status. In the Summer of 2016, Donald Trump was one of the few actors who considered 9/11 a Muslim event. Yet given the disruptive character of his presidency (Holland & Fermor, 2020; Lamont et al., 2017; Wagner‐Pacifici & Tavory, 2017), it is not unthinkable that he may have subsequently created consonance for this discourse. In fact, Trump's presidential era would be an ideal case to investigate the possibilities of the “unlocking” of the central event meanings—of 9/11, but also of other major national occurrences—within dominant American interpretive structures.

## CONFLICT OF INTEREST

None.

## Data Availability

The data (newspaper articles and legislative documents) come from publicly available website. The newspaper articles are downloaded from the LexisNexis databank (the specific newspapers and search terms are included in the article). The American legislative documents come from the website congress.gov, and the Dutch documents are derived from officielebekendmakingen.nl (the specific search terms are also included in the article).

## ENDNOTES

1 Kingdon’s conceptualization of focus events is not the sole prominent use of the term in event literature. Birkland (1997) and Alimi and Maney (2018) employ it later to refer to occurrences that attract significant attention (receive focus). Because in this article I examine social change and not attention, I use Kingdon’s definition.

2 The articles came from all national newspapers from the two countries that are downloadable through LexisNexis: *Daily News (New York), The New York Times, The Washington Post and USA Today* in the United States; and *Algemeen Dagblad, De Volkskrant, De Telegraaf, NRC Handelsblad and Trouw* in the Netherlands. The *Daily News's* main focus is local news (even though it writes extensively about national and international affairs). I included it in the data because I did not want to solely investigate elite newspapers for the American case.

3 Using this *search term* provided me with a database of 100,234 articles in total, of which 80,013 were American and 20,221 were Dutch. All other 9/11‐related search terms appeared to be less useful than ‘September 11’. ‘Twin Towers’ or ‘World Trade Center’ were not employed that often, except for the first months after the attacks. ‘9/11’ was a tricky search term as it included many sports statistics (‘9 vs. 11’) or time schedules (‘from 9 to 11’). Using this term would have taken quite some time of excluding non‐related articles. This might have been worthwhile if it had led to different research results. However, an exploratory investigation of articles with the search terms ‘September 11’ and ‘9/11’ indicated that these did not vary substantially.

4 All Dutch quotes have been translated by the author.

5 I make use of this source (an opinion magazine, not a newspaper) because it was the specific outlet in which Sylain Ephimenco published his “Open Letter” regarding the El Moumni affair.
